# A Retrospective Analysis of Diagnostic Breast Imaging Outcomes in Young Women at a Tertiary Care Center

**DOI:** 10.3390/curroncol31070291

**Published:** 2024-07-06

**Authors:** Navdeep Dehar, Doris Jabs, Wilma Hopman, Mihaela Mates

**Affiliations:** 1 Department of Oncology, Queen’s School of Medicine, Queen’s University, Kingston, ON K7L 5P9, Canada; doris.jabs@kingstonhsc.ca (D.J.); mihaela.mates@kingstonhsc.ca (M.M.); 2 Cancer Centre of Southeastern Ontario, Kingston Health Sciences Centre, Kingston, ON K7L 2V7, Canada; 3 KGH Research Institute, Department of Public Health Sciences, Queen’s University, Kingston, ON K7L 3N6, Canada; wilma.hopman@kingstonhsc.ca

**Keywords:** breast cancer, young women, screening, diagnostic mammogram, ultrasound

## Abstract

(1) Purpose: The purpose of this study was to describe the outcomes of diagnostic breast imaging and the incidence of delayed breast cancer diagnosis in the study population. (2) Methods: We collected the outcome data from diagnostic mammograms and/or breast ultrasounds (USs) performed on women between the ages of 30 and 50 with symptomatic breast clinical presentations between 2018 and 2019. (3) Results: Out of 171 eligible patients, 10 patients (5.8%) had BIRADS 0, 90 patients (52.6%) had benign findings (BIRADS 1 and 2), 41 (24.0%) patients had probable benign findings requiring short-term follow-up (BIRADS 3), while 30 (17.5%) patients had findings suspicious of malignancy (BIRADS 4 and 5). In the BIRADS 3 group, 92.7% had recommended follow-up, while in BIRADS 4 and 5, only 83.3% underwent recommended biopsy at a mean time of 1.7 weeks (range 0–22 wks) from their follow-up scan. Ten (6%) patients were diagnosed with breast cancer, all of whom had BIRADS 4 or 5, with a mean time of breast cancer diagnosis from initial diagnostic imaging of 2.2 weeks (range 1–22 wks). No patients had delayed breast cancer diagnosis in our cohort. (4) Conclusions: We conclude that diagnostic mammograms and breast US are appropriate investigations for clinical breast concerns in women aged 30–50 years.

## 1. Introduction

Breast cancer is the most commonly diagnosed malignancy among women worldwide, regardless of their age [1]. According to Canadian Cancer Statistics 2023, an estimated 29,400 Canadian women will be diagnosed with breast cancer, representing 26% of all new cancer cases in women in 2023 [2]. Although breast cancer is more common among elderly women, the incidence of breast cancer is on the rise in the younger population [2]. In the U.S. alone, there has been an average 2% annual rise in breast cancer cases between the ages 40 and 49 from 2015 to 2019 [3]. The 2% rise in breast cancer detection in this population is thought to be due to the increased adoption of screening in this age group [4]. Each year, more than 1000 women under the of age 40 die from breast cancer, having a 40 percent higher mortality rate and worse outcomes [5,6] than women over the age of 50. Screening for breast cancer is an effective measure for early detection, improving the survival rate and reducing the disease burden of cancer patients [7,8,9]. The majority of guidelines, including Canadian guidelines, recommend screening for breast cancer in women aged 50–69 [10,11,12,13]. However, although most breast cancer cases do occur in women older than 50 years of age, 23 percent of all breast cancer diagnoses occur in those under the age of 50 [14,15]. Most recently, given the rise in breast cancer diagnoses in young adults, the U.S. Preventive Services Task Force updated its screening guidelines to recommend that all women be screened for breast cancer every other year starting at age 40 [5].

Nearly 80% of breast abnormalities in young women are self-diagnosed, leading to late presentations and biologically more aggressive disease, with greater rates of recurrence and metastatic disease [2]. In addition, the prevalence of breast cancer in pregnant women and new mothers has also increased, making it the most common form of cancer in this population, with an estimated 30% or more receiving this diagnosis within a few years postpartum [7,16,17]. Multiple studies have shown that the overall sensitivity of mammography is only 80%; despite having a negative screening mammogram test, some patients would require more testing [17,18]. This is especially true for young women with dense breasts, who are at risk of potentially delayed diagnosis and poor outcomes [19]. Furthermore, applications of artificial-intelligence–based neural networks are being explored to enhance the efficiency of US and breast MRI for better and early detection of structural breast abnormalities [20].

The sensitivity of mammography depends on many factors including age, breast density (percentage of breast fat vs. other tissue), tumor type and growth patterns (lobular vs. ductal carcinomas), and technical/interpretive errors [21,22,23,24,25,26]. A retrospective analysis of the performance of screening and diagnostic mammograms in women younger than 40 showed high recall rates, along with a high frequency of additional imaging [19]. The sensitivity, specificity, and positive predictive value of the test were inadequate, and the cancer detection rates was meager [19]. Most recently, the 2023 Canadian Association of Radiologists Breast Disease Imaging Referral Guidelines also recommended additional complementary imaging in the form of targeted US and diagnostic mammography or digital breast tomography of the area of clinical concern as the initial imaging techniques [19].

The purpose of our study was to describe the outcomes of diagnostic mammograms and breast US used for the work-up of abnormal clinical breast abnormalities and to describe the frequency of delayed breast cancer diagnosis (more than six months after initial diagnostic mammography) in this population.

## 2. Materials and Methods

### 2.1. Patient Cohort

A retrospective electronic chart review was conducted at our institution, including all women between the ages of 30 and 50 years who underwent diagnostic breast imaging for clinical breast concerns such as breast pain, palpable mass, nipple discharge, or breast skin changes. These patients were assessed with diagnostic mammograms and/or breast ultrasound (US) at the Kingston Health Sciences Centre (Kingston, ON, Canada) between January 2018 and December 2019. Data were collected retrospectively in May 2022, with a median follow-up time from the first patient’s initial imaging to the time of data collection of 30.7 months, with a range 22.0–69.7 months. Institutional research ethics board approval was obtained prior to study initiation. We excluded all patients undergoing routine screening mammography and those with incomplete chart information.

Patient demographics, types of clinical breast presentations, type and timing of initial and follow-up breast imaging, findings of initial and follow-up breast imaging using the Breast Imaging Reporting and Data System (BI-RADS) and breast cancer diagnosis timelines, where applicable, were collected from the electronic patient records. In addition, percent mammographic breast density, a visual estimate of the percentage of fibroglandular (dense) tissue in the breast, reported as <75% or ≥75%, was documented. Where available, data on previous screening investigations were captured. The type of biopsy and pathology results were recorded for patients who underwent biopsy. In patients with a diagnosis of breast cancer, pathologic features and staging were collected from the electronic medical records (EMRs).

### 2.2. Outcomes

The primary outcome measure was the rate of delayed breast cancer diagnosis in this group, which was defined as a breast cancer diagnosis occurring more than 6 months after the initial diagnostic imaging.

Secondary outcomes included a description of the type of initial and subsequent examinations and their findings, completion of recommended follow-up tests, as well as the total number of breast cancer diagnoses and their respective stages.

### 2.3. Statistical Analysis

Data were collected in an Excel spreadsheet designed for the study, and imported into IBM SPSS (version 28.0 for Windows, Armonk, NY, USA, 2022) for statistical analysis. Clinical characteristics and findings of the diagnostic imaging were summarized using descriptive statistics including frequencies and percentages for categorical data and means with standard deviations, or medians with quartiles (as appropriate) for continuous data. The underlying distribution of the continuous data was assessed with the Shapiro–Wilk test. This study was purely descriptive; therefore, no inferential statistics or *p*-values are reported.

## 3. Results

### 3.1. Patient Characteristics

Of the 400 electronic patient charts reviewed, 171 were identified as eligible for data analysis. The common reasons for exclusion were incomplete data capture and patients with routine screening mammograms. The baseline patient characteristics are summarized in Table 1. The mean age of patients at the time of clinical presentation was 40.5 ± 5.7 years. The most common clinical presentations among the identified patients were palpable breast mass and breast pain.

### 3.2. Baseline Investigations

The initial breast imaging and findings are summarized in Table 2. Most patients (86.5%, *n* = 148) had both a diagnostic mammogram and a breast ultrasound (US) to evaluate the clinical breast concerns. Some patients (11.7%, *n* = 20) had breast US alone, while only 3 (1.8%) patients had a mammogram alone as initial investigation. Eleven (6.4%) patients had previous screening mammograms. Information about the breast density was not reported consistently on initial imaging during the study time frame. Only 20 (11.7%) patients in our cohort had breast density reported as ≥75%.

Concerning the findings of the initial imaging, more than half of the patients had benign findings (BIRADS 1 and 2), and about one-quarter had probable benign findings requiring short-term follow-up (BIRADS 3). In total, 17.5% (*n* = 18) of patients had findings suspicious of malignancy (BIRADS 4 and 5), with biopsy recommended for diagnosis (Table 2).

### 3.3. Follow-Up Investigations

The outcomes of the follow-up investigations are summarized in Table 3. For the patients who were recommended to have follow-up investigations (BIRADS 0, *n* = 10, and BIRADS 3 group, *n* = 41) after their initial imaging, all patients in BIRADS 0 and the majority (92.7%) in BIRADS 3 had the recommended follow-up breast imaging, and, for the remaining three patients, no follow-up information was available in the electronic records.

For the BIRADS 0 score, out of all patients who went for re-imaging, two patients were re-scored as BIRADS 3 and 4 in each category, requiring short-term follow-up and immediate biopsy, respectively. In the BIRADS 3 group, 78% had breast US as a subsequent investigation, of which 22.0% (*n* = 9) were benign lesions (BIRADS 1 and 2) and 68.3% (*n* = 28) remained in their BIRADS 3 category, while none were scored BIRADS 4 or 5.

All patients in the BIRADS 4 and 5 group (17.5%, *n* = 30) were recommended to have a diagnostic breast biopsy, of which 83.3% (*n* = 25) underwent the procedure at a median interval time of 3.7 weeks (Table 3). The remaining five patients did not undergo the recommended procedure despite the high risk of breast cancer malignancy. Out of the patients who underwent breast biopsy (83.3%, *n* = 25), 10 patients were diagnosed with breast cancer, representing (6%) out of the total 171 patients included in the study. Fifteen patients (8.7% of the entire enrolled population) had benign findings, including one biopsy with dysplastic changes requiring no further action. The median time interval between follow-up scan and biopsy was 1.7 weeks (range 0–22 wks).

### 3.4. Breast Cancer Diagnosis

Out of the total 171 patients included in the study, 10 patients (6%) were diagnosed with breast cancer upon further investigations, with a mean age of 38 ± 6.5 yrs (range 31–49). All 1-patients (100%, n = 10) had a diagnostic breast US, and 72.7% (n = 16) had diagnostic mammograms with BIRADS 4 or 5, while only 25% (n = 4) had a reported breast density of >75%. The stage distribution showed 80% (n = 8) had stage 1 and 20% (n = 2) had stage 2 disease. None of these patients were identified with locally advanced or metastatic disease (Table 4).

### 3.5. Delayed Breast Cancer Diagnosis

The primary outcome of the study was the frequency of delayed breast cancer diagnosis, defined as a breast cancer diagnosis occurring more than six months (24 weeks) after the initial diagnostic imaging. In our cohort, the median time from the initial imaging to breast cancer diagnosis (via biopsy) was 2.2 weeks, with an interquartile range of 1.1 to 7.2 weeks and a range of 1 to 22 weeks. Notably, none of the patients in our cohort experienced a delayed breast cancer diagnosis, as outlined in Table 4.

## 4. Discussion

Breast cancer is a significant global health concern. The recent global cancer statistics indicate that breast cancer incidence has surpassed that of lung cancer and is the most frequently diagnosed cancer worldwide, accounting for 11.7% of new cancer cases in 2020 [27]. According to the 2022 Global Breast Cancer Report, the overall median age at diagnosis is 62 years; however, the incidence of breast cancer is rising in young women (20–49 years) [28,29]. Early investigation of clinical breast concerns is important in young women because tumor behaviors are usually more aggressive than in their older counterparts, and delayed diagnosis causes a disproportionate number of life years lost [28]. In our study, we investigated the outcomes of diagnostic breast imaging and the frequency of delayed diagnosis of breast cancer in young women between the ages of 30 and 50 presenting with clinical breast concerns. Our study did not show any delayed breast cancer diagnoses, but these results should be interpreted with caution due to the small number of patients and short follow-up period. Most patients in our study with abnormal results at baseline, including those with a breast cancer diagnosis, underwent the recommended follow-up imaging/procedures in a timely manner. Most importantly, there were no breast cancer diagnoses in patients with normal/benign findings on original diagnostic imaging (BIRADS 1 and 2 categories). All patients with a breast cancer diagnosis had early-stage disease (Stage 1–2) and received the most appropriate curative treatment.

Mammography and ultrasound are the standard imaging techniques for detecting and evaluating clinical breast concerns such as breast mass, breast skin dimpling, breast or nipple pain, nipple retraction or nipple discharge, and enlarged lymph nodes [27]. When it comes to evaluating women with clinical symptoms, the selection of primary breast imaging is partly based on age [30]. However, little evidence exists supporting a specific age in determining the best choice of initial diagnostic breast imaging in symptomatic women. In the absence of definitive evidence, experts suggest that women younger than 35 years should be evaluated with ultrasound, and that women over 35 should undergo mammograms and US [31,32]. Breast ultrasound carries a higher sensitivity for detecting breast cancer in women with dense breast tissue, women under the age of 50, and high-risk women [33]. In our study, 93.6% of patients had their first diagnostic imaging. Out of these, more than 86% of patients had both a diagnostic mammogram and breast US for the first presentation of a clinical breast abnormality, showing that physicians are aware of the limitations of mammograms and current guidelines. This is consistent with the recent Canadian Radiologists Breast Cancer Referral guidelines [19] recommending mammography/digital breast tomography and targeted US of the area of clinical concern as the initial imaging in women 30 years of age and older with symptomatic breasts.

Breast MRI may be performed when breast cancer is suspected and/or other imaging studies have yielded equivocal results [34]. However, there is no literature available comparing mammography/breast US to MRI as part of the initial work-up of breast complaints. In a study of 1441 women with dense breasts who underwent routine screening, abbreviated breast MRI was associated with a significantly higher rate of invasive breast cancer detection compared with digital breast tomosynthesis [35]. Therefore, breast MRI may be the better alternative for work-up of women with dense breasts.

Breast cancer diagnosis delay is not only associated with reduced survival but also a greater risk of needing more aggressive treatments. In a study by Hutazuu et al., only 35.3% of patients presenting with breast symptoms had their breast cancer diagnosed within one month [31]. In our study, more than half of the patients had benign findings, and 15% needed an upfront biopsy. The median time between initial imaging and breast cancer diagnosis in our study was 2.2 weeks (range 1 to 22 weeks). In our cohort, we found that five patients in the BIRADS 4 and 5 groups, in which there was a high probability of breast cancer diagnosis, did not undergo the recommended follow-up investigations. Unfortunately, we do not have any information as to the reason. It may be due to a lack of follow-up to discuss results, miscommunication with regard to the significance of the results, and/or the patient’s refusal to undergo additional investigations. Such patients are at risk of missed/delayed diagnosis and poor outcomes, which underlines the need for adequate processes to disclose test results and their significance and to mitigate barriers to care. In another study by Ouyang et al., 26.8% of patients in the study cohort were lost to follow-up within the first five years of post-operative surveillance [32]. The plausible reasons highlighted were young age, distance between patients’ residence and the hospital, and medical insurance status (uninsured vs. insured). Similarly, in another study by Quyyumi et al., 21% of patients were lost to follow-up within 5 years of diagnosis [36]. We must raise awareness about system- and patient-related barriers and encourage women of all ages to take proactive steps toward early detection and prevention.

Contrary to previously published work [37,38,39,40], the pathology results of our study showed localized, early-stage, intermediate-to-high grade, and primarily hormone receptor (ER/PR)-positive tumors in this young age group who presented with clinical breast concerns. We did not find any worse prognostic characteristics than those described in older age groups (i.e., higher rates of lymph-node-positive disease or HER2+/TNBC) [29]. This is likely due to the low number of invasive breast cancer diagnoses. On the other hand, our study highlights the importance of appropriate and timely investigations in young patients, which result in early detection of invasive disease and ultimately prevent the need for more aggressive treatments if the diagnosis is delayed.

## 5. Limitations

The most significant limitation of this retrospective analysis is the small number of patients with a breast cancer diagnosis from a single, small cancer center. Data were collected from 2018 to 2019, during which time breast density information was not consistently reported in the initial diagnostic imaging. As highlighted in the study results for follow-up diagnostic investigations, only 20 (11.7%) patients had a breast density ≥ 75%. Breast density could have had implications on the number of missed breast cancer diagnoses that this study could not address. In addition, some patients in our study with high-risk features on initial imaging did not have recommended follow-up diagnostic investigations. Due to the limitations of follow-up data, we could not collect additional information as to the reasons for missed investigations or late breast cancer diagnoses in the patients lost to follow-up.

## 6. Conclusions

In our study, we describe the outcomes of diagnostic breast imaging in women 30–50 years old presenting with clinical breast concerns, the rate of completion of recommended follow-up investigations, the total number of breast cancer diagnoses, and the rate of delayed diagnosis. The results show that most patients presenting with breast symptoms had benign findings on initial breast imaging (mammogram and/or breast US). In contrast, only a small number ultimately had a diagnosis of early-stage breast cancer. There were no delayed breast cancer diagnoses in those with abnormal initial imaging, and, most importantly, no patients with normal initial imaging results had a diagnosis of breast cancer due to false negative results at the time of data collection. Therefore, we conclude that diagnostic mammograms and US are appropriate diagnostic investigations for clinical breast concerns in women between 30 and 50 years.

## Figures and Tables

**Table 1:** Baseline patient characteristics.

Patient Characteristic (n = 171)	n (%)
Mean age ± standard deviation at presentation	40.5 ± 5.7 y
Breast symptoms at the time of presentation	
Breast mass	87 (50.8)
Breast pain	67 (39.1)
Axillary lymph node/mass	5 (2.0)
Breast asymmetry	1 (0.06)

**Table 2:** Baseline investigations.

Baseline Investigations (n = 171)	n (%)
Baseline screening mammogram	11 (6.4)
Baseline Diagnostic Imaging	
Mammogram alone	3 (1.8)
Breast US alone	20 (11.7)
Mammogram and breast US	148 (86.5)
BIRADS Category	
BIRADS 0	10 (5.8)
BIRADS 1 and 2	90 (52.6)
BIRADS 3	41 (24.0)
BIRADS 4	24 (14)
BIRADS 5	6 (3.5)
Breast Density	
Not reported	20 (11.7)
<75%	131 (76.6)
75% or more	20 (11.7)

**Table 3:** Follow-up investigations.

Follow-Up Investigations	n (%)
BIRADS 0 Group Follow-Up Imaging (n = 10)	
Mammogram	1 (10.0)
MRI	6 (60.0)
Diagnostic mammogram and US	3 (30.0)
Results of BIRADS 0 Follow-Up Imaging (n = 10)	
BIRADS 0	1 (10.0)
BIRADS 1	5 (50.0)
BIRADS 3	2 (20.0)
BIRADS 4	2 (20.0)
BIRADS 3 Group Follow-Up Imaging (n = 41)	
Diagnostic US	32 (78.0)
Diagnostic mammogram and US	6 (14.6)
None	3 (7.3)
Results of BIRADS 3 Follow-Up Imaging (n = 41)	
BIRADS 0	1 (2.4)
BIRADS 1	4 (9.8)
BIRADS 2	5 (12.2)
BIRADS 3	28 (68.3)
Total	38 (92.7)
Missing	3 (7.3)
Biopsy for BIRADS 4 or 5 (n = 30)	
Yes	25 (83.3)
No	5 (16.7)
Biopsy Result for BIRADS 4 or 5 (n = 25)	
Malignant (invasive or noninvasive)	10 (33.3)
Benign	15 (47.0)

Median time between follow-up scan and biopsy was 1.7 weeks (IQR 1.2–3.2, range 0–22 weeks).

**Table 4:** Patients with breast cancer diagnosis.

Total Patients (n = 10)	n (%)
Mean Age (range 31–49 yr)	38.2 ± 6.5 y
Reported Breast Density ≥75%	2 (20)
Diagnostic Mammogram	
Yes	7 (70)
No	3 (30)
Diagnostic US	
Yes	10 (100)
Breast MRI	
Yes	2 (20)
Stage Distribution	
1	8 (80)
2	2 (20)
3	0 (0)
4	0 (0)
Invasive Tumor Grade	
1	2 (20)
2	5 (50)
3	3 (30)
Biomarker Status	
ER positive	9 (90), 1 missing
PR positive	9 (90), 1 missing
HER2 negative	8 (80), 2 missing
Breast Cancer Management	
Surgery (partial or full mastectomy)	10 (100)
Chemotherapy	2 (20)
Endocrine Therapy	9 (90)

Median time between initial imaging and breast cancer diagnosis was 2.2 weeks (IQR 1.1–7.2, range 1–22 weeks).

## Data Availability

The original contributions presented in the study are included in the article, further inquiries can be directed to the corresponding author.
